# Unrecognized perforation into the anterior interventricular vein complicating PCI for anterior STEMI: An unexpected detour

**DOI:** 10.1002/ccr3.4055

**Published:** 2021-05-05

**Authors:** Eric S. Rothstein, Jennifer Frampton, James T. Devries, Michael N. Young

**Affiliations:** ^1^ Dartmouth‐Hitchcock Medical Center Heart and Vascular Center Geisel School of Medicine Lebanon NH USA

**Keywords:** complication, coronary perforation, fistula, myocardial infarction

## Abstract

Large iatrogenic coronary artery perforations require rapid management; however, operators must be able to recognize guidewire perforation into cardiac veins in order to avoid causing further complications with standard salvage strategies.

## Graphical abstract

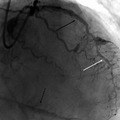

## 1 INTRODUCTION

Coronary artery perforation (CAP) is one of the most feared complications of percutaneous coronary intervention, with an estimated mortality approaching 20%.^1^, ^2^ Rarely, guidewire perforation into a cardiac chamber or another vessel has been reported and is classified as an Ellis type 3 cavity spilling perforation.^3^ We describe an unfortunate case of CAP during intervention for anterior ST‐elevation myocardial infarction (STEMI). The guidewire exited the left anterior descending (LAD) artery and perforated into the anterior interventricular vein (AIV). This was misdiagnosed as multiple guidewire perforations into the coronary sinus (CS), but with the belief that the wire had reentered the LAD. This was managed using covered stents, creating a large iatrogenic LAD‐AIV fistula.

**FIGURE 1:**
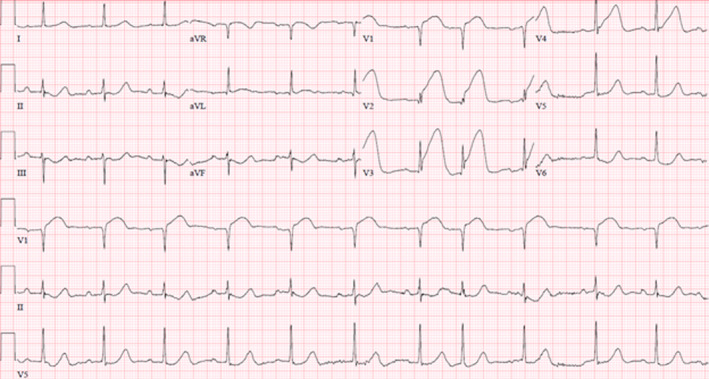
ECG demonstrating sinus rhythm with premature supraventricular complexes and 6 mm ST‐elevation in the anterior leads consistent with acute myocardial infarction

An 84‐year‐old man with history of end‐stage renal disease and remote kidney transplant (18 years prior) developed acute crushing chest pain and was brought to our emergency department by emergency medical services with a diagnosis of anterior STEMI based upon electrocardiogram (ECG) obtained in the field. Repeat ECG (Figure 1) confirmed the diagnosis.

**FIGURE 2:**
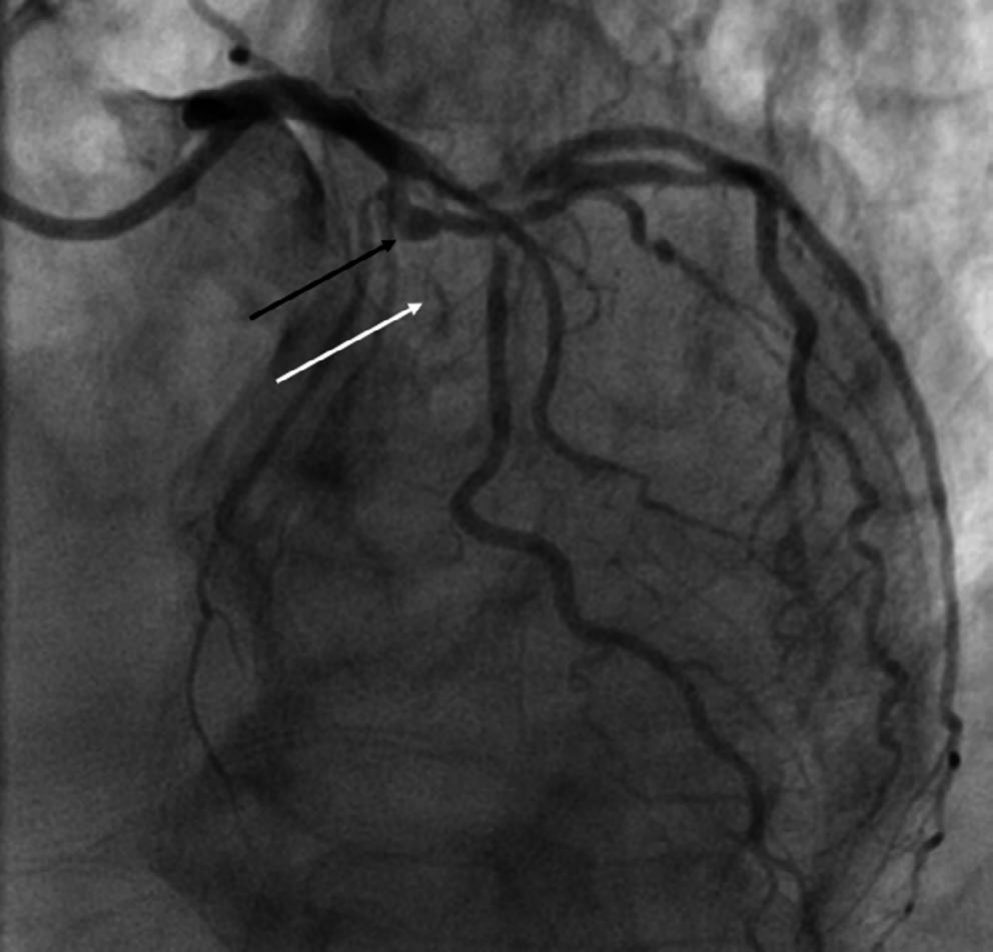
Initial angiogram taken from the LAO‐cranial projection demonstrating a mid‐LAD aneurysm with high grade occlusion (black arrow) and trace antegrade contrast flow into the distal vessel (white arrow)

On examination, he was hemodynamically stable but uncomfortable due to chest pain. He was taken to the cardiac catheterization laboratory for emergent coronary angiography. Angiography revealed severe three‐vessel atherosclerotic coronary artery disease (CAD), including nonculprit high grade obstructive lesions in the proximal right and mid‐circumflex coronary arteries, as well as a culprit complete occlusion of the mid‐LAD immediately distal to an aneurysmal segment of the vessel that also gave rise to a large diagonal branch (Video S1 & Figure 2).

## 2 CASE REPORT

Repeated attempts were made to primarily wire the LAD using a Runthrough workhorse wire (Terumo Medical); however, this was unsuccessful as the wire was constantly deflected into the diagonal branch (Video S4). Microcatheter‐supported wiring using a Fine Cross MG (Terumo Medical) was unsuccessful as well (Video S2).

Attempts at wiring the lesion using a hydrophilic tapered wire (Fielder‐XT; Asahi Intecc USA) and a polymer jacketed wire (Pilot 50: Abbott Vascular) supported by a stiffer microcatheter (Corsair; Asahi Intecc USA) also failed. An emergent multidisciplinary team meeting was called between a cardiac surgeon, the interventional cardiologist, and the patient's inpatient cardiologist. All physicians agreed that due to the patient's age, frailty, and comorbidities he was not a surgical candidate. The patient continued to experience severe chest pain despite aggressive analgesic administration with clear ST‐elevations still present on ECG. Given the extensive calcific disease appreciated on his angiograms, it was hypothesized that treating the lesion as a chronic total occlusion (CTO) and proceeding with an antegrade wire escalation strategy might prove successful. Ultimately, the lesion appeared to be crossed using a controlled drilling technique with a Miracle Brothers 3 wire (Asahi Intecc USA) (Video S3).

The microcatheter was advanced into the distal vessel and intraluminal position was confirmed via a distal tip injection (Video S4). Of note, in this high acuity situation, the operators' focus was mistakenly restricted to the fact that the wire appeared to be intravascular, and the unusual appearance of the vessel was not appreciated at this time. On retrospective review after the case, the distal vessel clearly displays “to‐and‐fro” flow with drainage into the coronary sinus along with medial and lateral branches that are different in appearance from typical LAD septal and diagonal branch vessels. These unrecognized findings are all hallmarks of the distal vessel being a cardiac vein (specifically the AIV), rather than the LAD.

As the operators believed that the microcatheter had crossed into the distal LAD, the distal vessel was then safely wired using a workhorse wire to allow for equipment delivery. Due to challenging equipment delivery, a 1.5 × 12 mm compliant balloon was used to predilate the lesion followed by a 2.0 × 15 mm compliant balloon. A subsequent angiogram revealed extensive contrast extravasation from the vessel leading to the diagnosis of CAP (Video S5).

**FIGURE 3:**
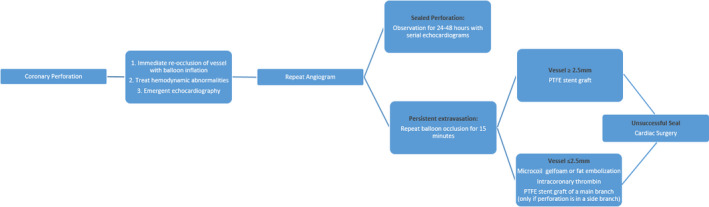
Suggested algorithm for management of coronary perforation (Modified from Textbook of Interventional Cardiology. Eng, Marvin H.; W. Moses, Jeffrey; Teirstein, Paul S. Published December 31, 2019. Pages 488‐506.e5. © 2020)^13^

Salvage techniques for CAP were initiated according to the standard perforation care algorithm detailed in Figure 3,^4^ although protamine administration was deferred.

Balloon tamponade was attempted utilizing the 1.5 mm balloon already in the vessel, followed by immediate upsizing to a 2.0 mm balloon that was inflated in the proximal vessel, with repeat angiograms showing no persistent contrast extravasation. Transthoracic echocardiography was performed, revealing no evidence of a pericardial effusion. Repeat injections performed during transient balloon deflations revealed continued extravasation of contrast. Obtaining an additional arterial access site for the purpose of utilizing dual‐guide catheter (“ping‐pong guides”) technique was discussed; however, this was deferred given the previous challenge crossing the lesion initially.

At this time, the patient developed vomiting, hypotension, and ultimately ventricular fibrillation requiring multiple defibrillations and chest compressions. He was intubated emergently and cardiac anesthesia performed transesophageal echocardiography, which showed no evidence of pericardial effusion, but an akinetic anterior wall of the left ventricle. An intra‐aortic balloon pump (IABP) was simultaneously inserted through the right femoral artery, and the patient was started on norepinephrine with resultant stabilization of his hemodynamics. Selective LAD angiography performed through a guide catheter extension demonstrated phasic flow in the distal vessel as well as a channel connecting the mid‐vessel and CS, which was thought to represent an iatrogenic arteriovenous fistula (AVF) (Video S6). The operators believed the etiology for these findings to be two separate wire perforations, with one perforation into the coronary sinus and one into the pericardial space.

Advancement of a covered stent across the mid‐vessel was not possible, so a 2.5 × 20 mm drug eluting stent (DES) was advanced and deployed across the perforation to permit tracking of a covered stent. Angiography following DES deployment revealed TIMI 3 flow in the distal vessel for the first time during the case, with continued robust flow into the CS through multiple channels. Additionally, an unusual “myocardial blush pattern” was observed as well as extensive flow into the coronary veins (Video S7).

A 2.8 × 16 mm Graftmaster covered stent (Abbott Vascular) was deployed across the visualized perforation; however, a residual channel connecting the vessel and the CS was visualized distal to the covered stent (Video S8).

**FIGURE 4:**
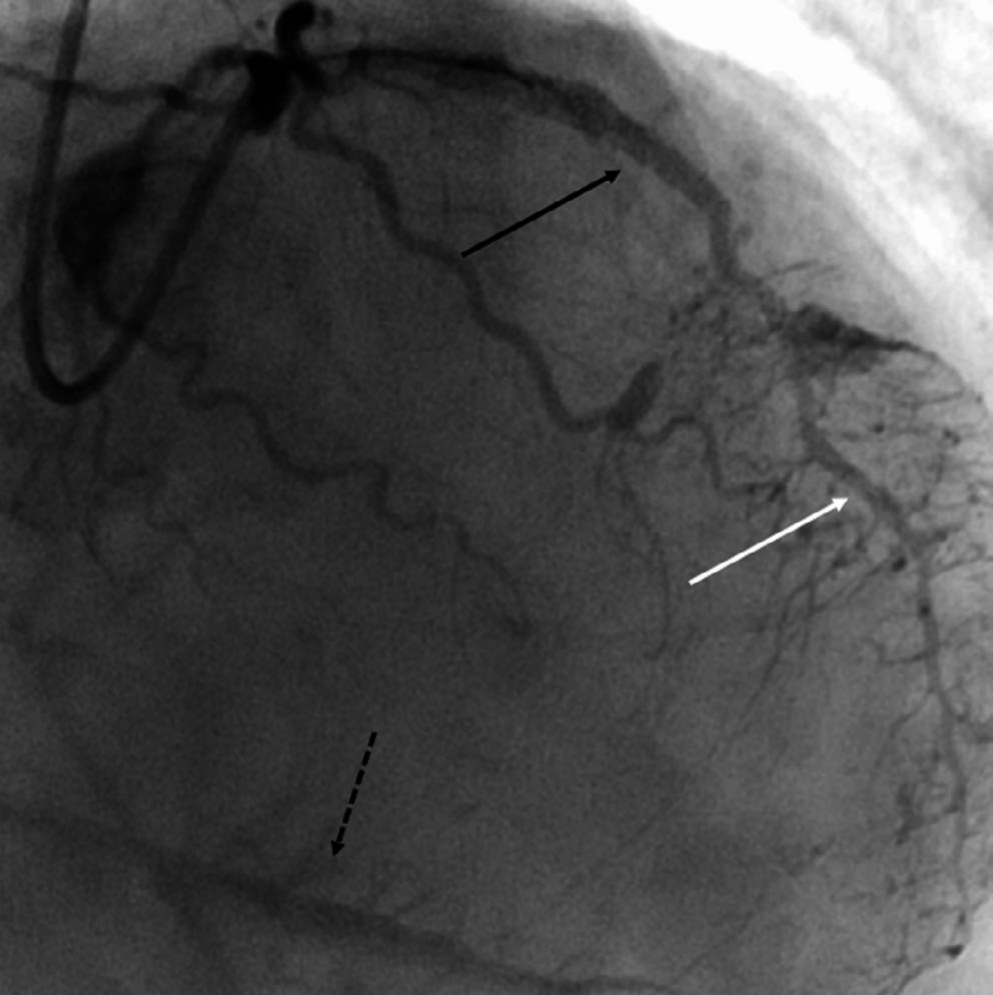
Completion angiogram taken from the RAO‐cranial projection demonstrating covered stent graft deployment (solid black arrow) with flow into the AIV and unusual stippling pattern (white arrow) with robust filling of the cardiac veins (dashed black arrow)

A second 2.8 × 16 mm covered stent was placed across the remaining channel and postdilated, effectively closing the perforation with no further flow visualized into the CS. Final angiograms also revealed several small contained wire perforations, persistence of the unusual “myocardial blush pattern” and even more robust filling of the major cardiac veins (Video S9 & Figure 4).

Post–procedure LV systolic function remained severely diminished, with an ejection fraction of 15%. He remained hemodynamically stable with an IABP in place on low dose norepinephrine and dobutamine. He went on to develop oliguric renal failure, and after multiple discussions with his wife surrounding goals of care, comfort measures were pursued and he died due to cardiogenic shock on post–procedure day four.

Upon dedicated review of the patient's images by a team of multiple interventional cardiologists, it was determined that the operator's original hypothesis that the wire had perforated into the CS multiple times prior to reentering the LAD was likely incorrect. It appeared that the stiff wire had perforated through the aneurysmal segment of the mid‐LAD into the AIV. A large AVF was created once angioplasty was performed across the perforation and was then propagated by the DES and ultimately the covered stent. The channel connecting the vessel to the LAD was the normal outflow from the AIV, which when a covered stent was deployed across, resulted in vigorous flow from the AIV into the other major cardiac veins. The “unusual blush pattern” is occasionally noted in selective pressurized cardiac vein injections typically performed during electrophysiology procedures.

## 3 DISCUSSION

Coronary artery perforation represents a rare, but potentially fatal complication surrounding PCI^5^ with the most common etiology being guidewire manipulation into the extravascular space.^6^ Management strategies for treating CAP must rely first upon hemodynamic stabilization.^7^ The vast majority of the published literature focuses on recognition and treatment of bleeding into the pericardial space. There are rare case reports surrounding perforation into a cardiac chamber or vein published.^3^, ^8^, ^9^, ^10^, ^11^ To the best of our knowledge, this is the first published case of a wire perforating into a cardiac vein misdiagnosed as a pericardial space perforation, with standard salvage techniques leading to the formation of a large AVF. There were several unique factors that predisposed this patient to the development of this complication, including a completely occluded vessel immediately distal to an aneurysmal arterial segment. In addition, the weakening of the vessel wall in combination with the use of a microcatheter‐supported 3 g tip‐load wire facilitated the wire's exit from the LAD and re‐entrance into the AIV. The unusual appearance of the vessel along with its unusual flow pattern was unrecognized by the operators in the setting of a patient in extremis. Had this flow pattern been recognized following microcatheter distal tip injection, the operators could have turned their attention back to primarily wiring the LAD versus pivoting to intravascular ultrasound (IVUS)‐guided re‐entry into the distal LAD in an effort to not only manage the fistula, but restore antegrade flow to the LAD.^10^ In the setting of these confusing angiographic signs, intravascular imaging with IVUS or optical coherence tomography would have likely clarified the location of the wire and identified perforation into the vein. Unfortunately, once the covered stents were deployed there is no percutaneous salvage strategy. The only way to potentially resolve the situation following stent graft deployment would be to proceed to emergency coronary artery bypass grafting, followed by occlusion of flow into the fistula.

## 4 CONCLUSION

Cases of CAP leading to iatrogenic AVF are very rare, and if unrecognized carry a grim prognosis. Unfortunately, the literature surround management of PCI‐related iatrogenic AVF is limited, and the optimal management strategy for perforation into a vein is not well defined.^12^ Understanding the risk factors that predispose a patient to perforation into a parallel vein, as well as meticulous attention to flow patterns in vessels is paramount to prompt recognition and appropriate management. CAP is a fearsome complication to manage acutely due to the potential for rapid decompensation of the patient as well as the complex technical challenges required for endovascular salvage therapy. An understanding of the angiographic appearance of the different Ellis classifications of CAP and familiarity with the unique bailout strategies for each subtype is of the upmost importance for all interventional cardiologists.

## CONFLICT OF INTEREST

None declared.

## AUTHOR CONTRIBUTIONS

ER: drafted and wrote the article. JF: edited and reviewed the article. JD: reviewed the article and provided key clinical insights. MY: revised the manuscript and helped prepare it for final submission.

## ETHICAL APPROVAL

This case is anonymous and was published with permission from the patient's family.

## Supporting information

Video S1

Video S2

Video S3

Video S4

Video S5

Video S6

Video S7

Video S8

Video S9

Supplementary Material

## Data Availability

All original angiograms (deidentified) are available at reasonable request.
