# Supplementary material for: Unrecognized perforation into the anterior interventricular vein complicating PCI for anterior STEMI: An unexpected detour
Source: Clin Case Rep. 2021 May 5;9(5):e04055. doi: 10.1002/ccr3.4055 (PMC8142412; doi:10.1002/ccr3.4055)
Supplement: Supplementary file 10 — Supplementary Material [file CCR3-9-e04055-s006.docx]

Video S1: Select initial diagnostic coronary angiograms revealing the culprit lesion in the mid-LAD

Video S2: Attempted initial primary wiring of the LAD followed by microcatheter-supported wiring

Video S3: Wiring of the distal vessel

Video S4: Microcatheter advancement into the distal vessel followed by distal tip injection

Video S5: Contrast extravasation following angioplasty of the LAD

Video S6: Selective injection of the LAD through a guide extension catheter following resuscitation, with phasic flow appreciated in the distal vessel and vigorous coronary sinus filling

Video S7: Following DES deployment, robust contrast extravasation into the coronary sinus with phasic flow in the distal vessel is appreciated

Video S8: Following initial covered stent deployment, a residual channel is seen connecting the vessel and the coronary sinus with exaggerated filling of the cardiac veins and an unusual “myocardial blush pattern”

Video S9: Completion angiograms following deployment and postdilation of a second covered stent demonstrating TIMI 3 flow through the vessel, contained wire perforations in small branch vessels, unusual stippling pattern, and forceful coronary vein filling
